# Mobile and pervasive computing technologies and the future of Alzheimer’s clinical trials

**DOI:** 10.1038/s41746-017-0008-y

**Published:** 2018-01-25

**Authors:** P. Murali Doraiswamy, Vaibhav A. Narayan, Husseini K. Manji

**Affiliations:** 1 Neurocognitive Disorders Program, Department of Psychiatry, Duke University Health System, and the Duke Institute for Brain Sciences, Durham, NC 27710 USA; 2 Janssen Research & Development, LLC, Raritan, New Jersey 08869 USA

**Keywords:** Medical research, Business and industry

## Abstract

The rapid growth of mobile phones, automated speech recognizing personal assistants, and internet access among the elderly provides new opportunities for incorporating such technologies into clinical research and personalized medical care. Alzheimer’s disease is a good test case given the need for early detection, the high rate of clinical trial failures, the need to more efficiently recruit patients for trials, and the need for sensitive and ecologically valid trial outcomes.

## Introduction

Alzheimer’s disease (AD) affects an estimated 45 million people worldwide, and despite substantial research investments, there are no therapies to prevent or slow disease progression.^1^ The 99.6% failure rate in recent AD clinical trials highlights the need for innovation and efficiency.^2^ It is believed that the best chance to intervene therapeutically is by targeting the preclinical or prodromal stages of Alzheimer’s. Some of these challenges may potentially be amenable to technological solutions.

The revolution in mobile technologies such as smart phones/tablets, cloud-based platforms, and deep learning-driven software algorithms and miniaturized automated physiological sensors is poised to profoundly disrupt medicine^3–7^ and, by extension, many aspects of neuropsychiatry and AD care and clinical research.^5–8^

In this Perspective, we highlight four areas in which such technology could facilitate AD clinical trials: (1) Mobilizing recruitment, (2) Mobile cognitive measures, (3) Digital functional outcomes, and (4) Integrated informatics platforms. For further information on how technology may help with care and caregiver support, readers are referred elsewhere.^4,5^

## Mobilizing clinical trial recruitment

ClinicalTrials.gov lists >450 active Alzheimer’s trials needing some 70,000 volunteers—posing the challenge of how to efficiently identify, consent, and screen subjects. Prevention trials increasingly rely on expensive brain scans or spinal fluid biomarkers to identify at-risk subjects but such methods have high screen fail rates—because only about a third of asymptomatic subjects may test positive.^8^ Thus time for subject recruitment may take up 30% of drug development costs and delay trials by ≥2 years.^8,9^

Brain health registries are one such solution wherein at-risk subjects are registered, self-consented, and prescreened via cognitive or even genetic self-tests. While academic AD centers have always had registries, the emergence of national registries could be a catalyst. The European Prevention of Alzheimer’s Dementia (EPAD) initiative, funded by the Innovative Medicines Initiative and the European Federation of Pharmaceutical Industries and Associations, plans to register 24,000 people to identify a Europe-wide cohort of >6000 high-risk participants, of which 1500 will be invited to participate in a technology-enabled trial to test new treatments for the prevention of AD.^10^ In the US, the Alzheimer’s Association TrialMatch (https://trialmatch.alz.org/find-clinical-trials#createaccount) allows caregivers and subjects to customize their search for trials and receive alerts. The Brain Health Registry (BHR) at USCF is pioneering a broad-based internet-based approach for recruiting and monitoring individuals at risk for AD.^11^ The registry is currently being used for pilot validation studies of mobile cognitive tests, and ultimately, all subjects enrolled in a large national biomarker trial (ADNI-3) will be given the option to participate in BHR.^11^ The Dominant Inherited Alzheimer’s Network (DIAN) and the Alzheimer’s Prevention Initiative (API) registries have successfully recruited subjects with specified genetic mutations. The Human Cognition Project (with >40 million subjects of all ages from 180 countries) provides a model for the kind of global brain laboratory that can be harnessed using mobile tools. This registry was recently used successfully to recruit and conduct a purely online randomized clinical trial of cognitive training.^12^

Research apps such as Apple’s Research Kit now also allow participants a simple way to consent, participate, and share their data,^13^ and these apps can be adapted for consenting caregivers and legal representatives. Ultimately, it is hoped such registries do not exist in silos, are able to recruit samples representative of the population, and keep subjects engaged over long periods to minimize selection and attrition biases.

## Mobile cognitive outcomes

A second challenge is that small, but clinically meaningful, treatment effects on cognition are often difficult to measure in preclinical AD or mild cognitive impairment (MCI) due to learning effects, ceiling effects, heterogeneity, and normal fluctuations. Composite endpoints formed by combining scores from existing neuro-psychological batteries have been proposed as primary endpoints for such trials. For example, the Alzheimer’s Disease Cooperative Studies-Preclinical Alzheimer’s Cognitive Composite, the primary outcome measure in the Anti-Amyloid Treatment in Asymptomatic Alzheimer’s study, is a composite of episodic memory, list learning, digit symbol substitution, and the Mini Mental State Examination.^14^ While it has shown promise in retrospective analyses of observational studies, it has not yet been fully validated.

**Fig. 1:**
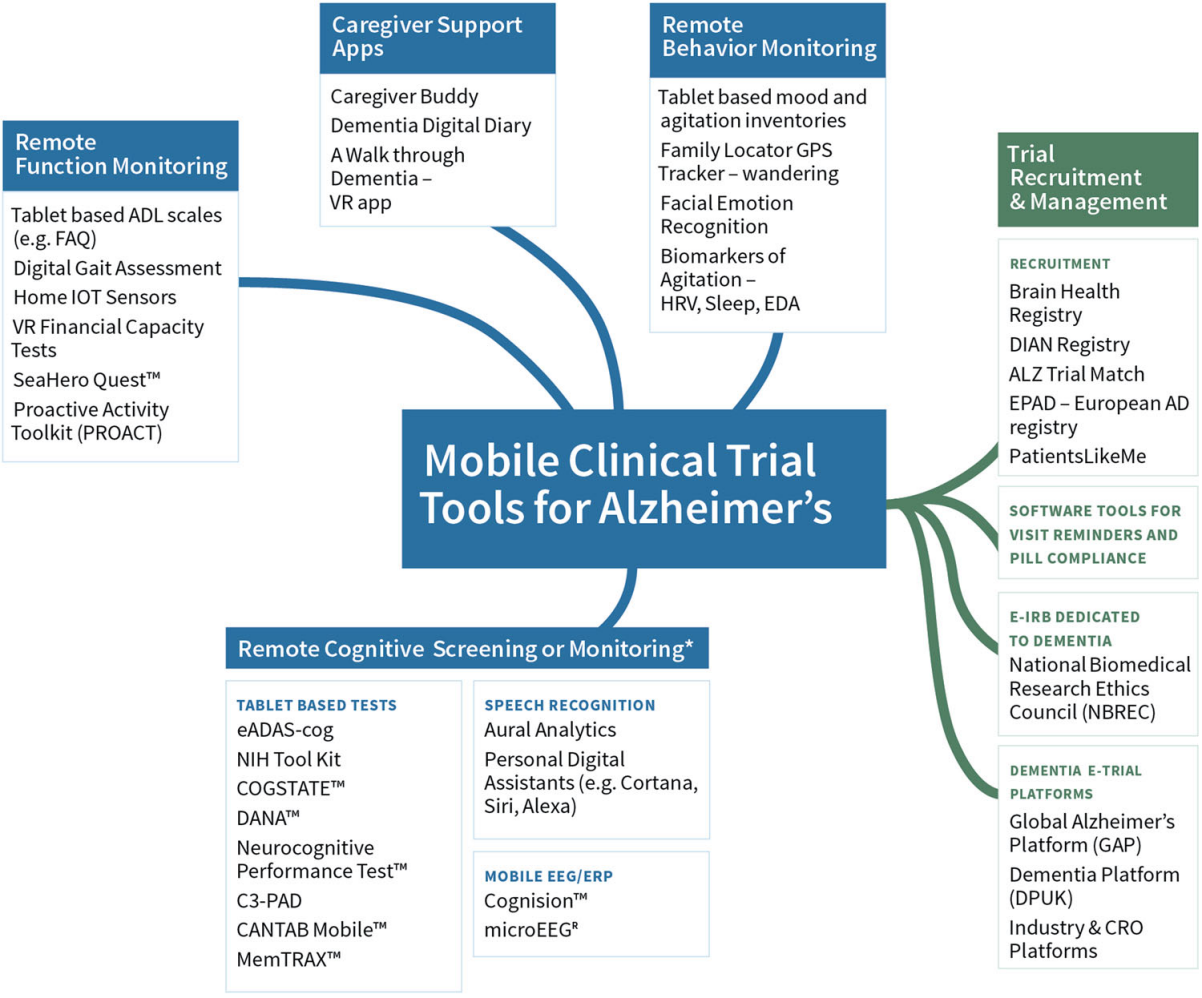
Figure depicts selected mobile or pervasive computing technologies that are being studied for use in AD research with examples in each area. This is not intended to be a comprehensive listing and the degree of validation that each technology has undergone is variable. The examples are intended to merely provide readers an overview of the developments in the field. HRV heart rate variability, ADAS refers to Alzheimer’s Disease Assessment Scale, EDA refers to electrodermal activity, FAQ refers to functional activities questionnaire, VR refers to virtual reality, other abbreviations are listed in the text

Automated mobile cognitive tests may offer advantages such as individualized scaling, automatic and error free scoring, and multiplicity of test versions and, if combined with gaming elements, may be less anxiety provoking. Many neuro-psychological tests and self-rated measures of cognition have been adapted for use on a tablet or phone, undergone varying degrees of qualification or validation (Fig. 1), and are already used in research and clinical trials. For example, a recent study of a iPad based self-test called C3-PAD (Computerized Cognitive Composite for Preclinical Alzheimer’s Disease) in 49 cognitively normal elderly subjects (mean age 71 years, 20% non-Caucasian) showed high reliability among the test versions (Cronbach alpha coefficient = 0.93). In all, 98% of subjects completed four out of five sessions correctly, and there was a high correlation between in-clinic and at-home C3-PAD assessments (*r* ^2^ = 0.508, *p* < 0.0001), suggesting promise for clinical trial use.^15^ The relationship with standardized tests covering similar cognitive domains was also significant but less robust (*r* ^2^ = 0.168, *p* < 0.003), suggesting that the tests are in need for some refinement.^15^ A number of other large epidemiological studies (e.g., Framingham study) and clinical trials have also incorporated mobile cognitive tests as exploratory outcomes. ADNI-3, a national biomarker study, is directly comparing an at-home computerized cognitive self-test measure vs. standard paper and pencil tests as well as pathological markers of AD progression in a sample of 1200 subjects ranging from normal to MCI to mild AD dementia.^11^

## Mobile assessment of function (activities of daily living) and behavior

Another key challenge in Alzheimer’s prevention trials is to demonstrate an impact on function (daily activities of living) in populations that have minimal to no functional deficits. Longitudinal studies have charted the timeline of functional losses from preclinical stage to MCI to AD and many activities of daily living (ADLs; e.g., spatial navigation, financial calculations, using phone or computer, driving) can be measured using phone sensors, GPS, wearables, or home sensors. For example, in a 6-week study of the Proactive Activity Toolkit (PROACT), investigators tagged objects in a home with 108 radiofrequency identification tags and analyzed signals by a prototype glove worn by a group of healthy adults. PROACT accurately identified the performance of an ADL in >80% of subjects and the specific ADLs in 73% of cases.^16^ Another study, CART, has installed strategically placed sensors in >480 homes of seniors and has been continuously monitoring gait, mobility, sleep, and activity for >10 years to develop signatures of cognitive and functional decline (http://www.ohsu.edu/xd/research/centers-institutes/orcatech/index.cfm). A number of previously validated paper and pencil functional scales are also now available in online or mobile versions. Electronic organizer tools such as the Google Calender and AP@LZ have been tested in proof-of-concept studies with small samples to ameliorate functional and memory deficits,^17–20^ which in turn may improve trial compliance.

Many behavioral changes seen in AD, such as wandering, sleep, circadian rhythm changes, depression, and agitation, can also potentially be tracked through use of mobile devices and sensors (Fig. 1). For example, the outcome of a 4-week phase-2 clinical trial of a dual orexin receptor antagonist in mild-to-moderate AD patients (*N* = 125) with irregular sleep wake rhythm disorder is sleep efficiency and wake efficiency measured using actigraphy through a wrist device (https://clinicaltrials.gov/ct2/show/NCT03001557). Another pilot study evaluated the utility of a wrist sensor (Philips DTI-2) and digital dashboard to track agitation and stress in six nursing home patients with dementia over 2 months (total recorded time was 142 h across 37 days).^21^ Wrist sensor data (galvanic skin conductance, accelerometry, skin and environment temperature, and ambient light) were extracted weekly and compared with 24 h observations made by nursing staff study across four parameters—sleep, aggression, stress, and normal. These data allowed the authors to develop objective thresholds with sensor data for defining “stress” and “agitation” in AD patients and develop a dashboard that allows a clinician to run a stress analysis for a given patient over a given time period.

Other researchers are studying the utility of a wearable camera^22^ and non-immersive virtual environments^23^ to detect everyday changes in function or behavior. For example, one study compared the performance of 24 subjects with mild-to-moderate AD vs. 32 normal controls on a laptop-based virtual coffee making task in a virtual reality kitchen. AD patients performed worse than controls on the virtual test, and their errors were correlated with both scores on standardized functional tests and caregiver ratings of their impairments.^24^ Such tools may in future offer promise due to their ecological validity and potential to provide evidence to payers of real-world outcomes.

## Cloud-based analytic platforms and trial networks for mobile AD trials

The fourth challenge is one of data sharing and bioinformatics. The vast majority of raw data from Alzheimer’s trials, even those funded by government agencies, are not readily accessible to the wider scientific or public community. One of the exceptions has been ADNI, which has made data sharing a priority from day 1 and led to >800 publications.^11^ Today, many AD-related informatics algorithms are trained using ADNI data, but there is no equivalent public resource for replication or validation. Further, databases created over the past two decades for AD trials may lack contemporary features, such as a patient portal, integrated sensor and smart phone data, patient engagement tools, and secure data sharing. Most such data platforms exist in silos with limited or no cross-integration ability. The Global Alzheimer’s Platform (GAP) network in the US^8^ and Dementia Platform in the UK (DPUK) (https://www.dementiasplatform.uk/about) are examples of some recent efforts to overcome these limitations. The WeCareAdvisor, a web-based clinical research platform aimed at interventions for behavioral disturbances in in dementia, is being tested in a randomized trial.^25^ The NIH will soon fund a clinical trials network and coordinating center for mobile cognitive trials (https://grants.nih.gov/grants/guide/rfa-files/RFA-AG-18-012.html).

## Evidence gaps and challenges of mobile technologies for AD trials

Technology comes with both promises and limitations. New dementia mobile technologies often undergo initial feasibility and acceptability testing but are rarely subject to rigorous randomized trials comparing them to traditional methods. There is also lack of regulatory clarity over how biometric and digital data should be applied in late-stage registration trials. Lack of interoperability of devices, operating systems and platforms, privacy, hacking risks, access, and ethics of sharing biometric data are examples of some other limitations. Further, uptake of smart phone digital technologies by some elderly subgroups (such as those on fixed incomes) remains low. And despite large numbers of people expressing initial interest in registries or apps, due to large drop outs only a small fraction may continue over time. Clinicians and CROs who conduct AD trials may not be well informed about the technical limitations of mobile devices requiring the building of new partnerships with engineers. Last but not least, regulatory and ethical guidelines often lag behind the rapid pace at which technology is evolving. Groups such as the Software as a Medical Device International Regulators Forum, Mobile Clinical Trials subcommittee of the Clinical Trials Transformation Initiative, and the ISTAART Technology PIA (https://act.alz.org/site/SPageServer?pagename=ISTAART_PIA_Technology) are addressing some of these challenges. The pilot digital health technology precertification program (https://www.fda.gov/MedicalDevices/DigitalHealth/UCM567265) announced by the U.S. Food and Drug Administration may also provide greater regulatory clarity.

## Conclusions

Given the promise of mobile technologies across multiple areas of AD clinical trials, we call for a more rapid systematic validation and a multi-stakeholder public–private partnership—involving caregivers and patients, academia, clinicians, industry, regulators, ethicists—to develop a framework for the optimal deployment of such tools. Technology is advancing rapidly and automatic speech recognition personal assistant devices, powered by artificial intelligence, may be game changers with regards to how the elderly will interact with devices in the future. Ultimately, it is hoped that such innovations will accelerate the testing and development of effective therapies to delay the onset of AD.
